# Inflammatory Myofibroblastic Tumor With Rapid Recurrence and Distant Metastasis: Report of a Rare Case

**DOI:** 10.7759/cureus.52069

**Published:** 2024-01-10

**Authors:** Dipti Kalita, Ruchi Rastogi, Gunmala Bhatnagar, Kunjahari Medhi, Sanjay K Pandey

**Affiliations:** 1 Laboratory Medicine, Histopathology and Cytopathology, Batra Hospital and Medical Research Centre, New Delhi, IND; 2 Laboratory Medicine, Histopathology and Cytopathology, Batra Hospital and Medical Research centre, New Delhi, IND; 3 Medical Oncology, Batra Hospital and Medical Research Centre, New Delhi, IND; 4 Cardiac/Thoracic/Vascular Surgery, Batra Hospital and Medical Research Centre, New Delhi, IND

**Keywords:** histomorphology of inflammatory myofibroblastic tumor, brain metastasis, recurrence, alk-inhibitor, inflammatory myofibroblastic tumor (imt)

## Abstract

Inflammatory myofibroblastic tumors (IMTs) are rare spindle cell tumors clinically, morphologically, and genetically heterogeneous, mimicking many other reactive and neoplastic lesions and creating great diagnostic problems. Although it is generally characterized by oncogene-derived proliferation of myofibroblasts in a background of polyclonal inflammatory cell infiltrates, morphological variations do occur requiring immunohistochemistry and molecular genetics to confirm the diagnosis. It encompasses a wide age range, and locations, mostly said to be of intermediate grade having a low risk of recurrence and metastasis. However, its biological behavior and course are variable and unpredictable.

Here, we report a case of thoracic IMT in a 32-year-old adult female presenting with a history of fever, cough, and chest pain associated with neutrophilic leukocytosis. Radiological investigations revealed a large mass in the thoracic region with possibilities of hydatid cyst and neurogenic tumor. Initial core needle biopsy specimen and subsequent local resection specimen revealed the diagnosis of IMT on histopathology and immunohistochemistry, having conventional morphology with expression of Anaplastic lymphoma kinase (ALK) protein. The patient developed rapid local recurrence and was started with first-generation ALK inhibitor Crizotinib. After a brief period of response, she developed vertebral and brain metastasis within a short span of time and was switched to a third-generation ALK inhibitor, Lorlatinib. The patient is on regular follow-up, has stable disease, and maintains a good quality of life after two years of diagnosis.

## Introduction

Inflammatory myofibroblastic tumors (IMTs) are rare spindle cell neoplasms of intermediate grade, frequently being confused with numerous inflammatory and reactive simulators till recent years. It has now been confirmed that these are oncogene-derived neoplasms with a low risk of recurrence and metastatic potential, composed of myofibroblastic and fibroblastic spindle cells.

They are clinically, morphologically, and genetically heterogeneous, mimicking many other reactive and neoplastic lesions creating a great diagnostic dilemma. They require genetic studies not only for diagnosis but for therapeutic and prognostic purposes also. Their behavior is unpredictable, and the morphology and genetic characteristics usually do not correlate with the future course of the disease.

Complete surgical removal of localized tumors remains the mainstay of treatment. Treatment of IMT is not yet standardized for locally advanced, recurrent, and metastatic disease, where radiotherapy, chemotherapy, and targeted therapy are considered, depending upon the extent of the disease. Here, we report a case of thoracic IMT occurring in an adult female presenting initially with a challenging diagnosis and later with rapid multiple recurrences and distant metastasis.

## Case presentation

A 32-year-old female was referred to pulmonary and hematology outpatient departments with a history of fever, cough, and chest pain associated with neutrophilic leukocytosis found on routine blood examination. Physical examination was unremarkable.

**Figure 1:**
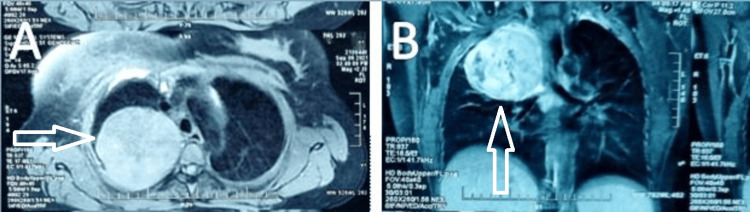
CT images of the tumor at the time of diagnosis CT scan thorax showing a well-circumscribed tumor (arrow) in the upper lobe apicoposterior segment, right lung at diagnosis. (A) Axial view, (B) sagittal view.

A routine blood examination revealed a white blood cell (WBC) count of 101x10^3^/µL with 82% neutrophils, hemoglobin 9.4g/dL, and platelet count of 430x10^3^/µL. Chest x-ray revealed opacities in the upper zone in the right lung. Noncontrast computed tomography (NCCT) thorax was done and a large well-rounded lesion measuring 7.0x8.3x7.8cm was revealed in the right upper lobe apicoposterior segment with surrounding reactionary change in lung parenchyma (Figures 1A, 1B). A provisional diagnosis of a hydatid cyst or a neurogenic tumor was made.

Magnetic resonance imaging (MRI) chest revealed a large well-defined heterogeneously enhancing mass lesion measuring 8.2x8.9x8.4cm in the right posterior paravertebral region with a broad base towards the mediastinum. Post-contrast, the lesion shows intense enhancement with central non-enhancing areas suggesting necrosis. Lung fields were clear, and minimal pleural reaction was seen. No evidence of erosion in the ribs was seen. The possibility of a neurogenic tumor was considered.

CT-guided trucut biopsy revealed a spindle cell tumor with minimal nuclear atypia, occasional mitotic figure, and necrosis. On immunohistochemistry (IHC), the spindle cells showed reactivity for Anaplastic lymphoma kinase (ALK) protein (SP144), and focally smooth muscle antigen (SMA) with a 15% Ki 67 labeling index, leading to the diagnosis of an IMT. The spindle cells were negative for S 100, SOX10, CD 34 and desmin, and thus neurogenic tumors, solitary fibrous tumors, and myogenic tumors were ruled out.

**Figure 2:**
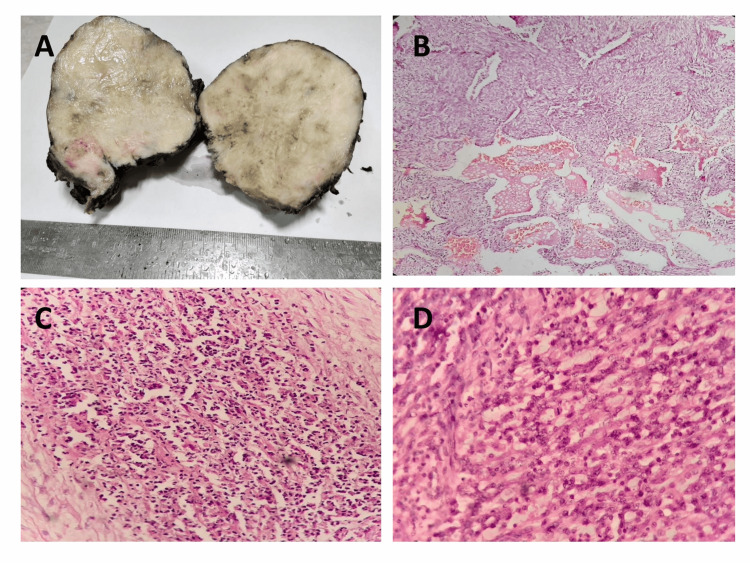
Gross and microscopic features of the tumor (A) Gross picture: well-circumscribed nodular tumor with solid, grey-white, fleshy partly myxoid cut surface. (B) Low power view of tumor, infiltrating lung alveolar spaces, H&E, x100. (C) Tumor showing neutrophilic collections, H&E, x400. (D) Tumor comprising fascicles of spindle cells infiltrated by inflammatory cells with occasional mitosis, H&E, x400.

The patient underwent right posterolateral thoracotomy with complete excision of the tumor. A 10x10cm size well-circumscribed mass adherent to the anterolateral surface of the upper lobe of the right lung was excised. The mass was received in two fragments, encapsulated and lobulated; the cut surface was grey-white, solid, homogeneous, and mostly fleshy with patchy myxoid areas (Figure 2A).

Hematoxylin and eosin (H&E) stained sections revealed interlacing fascicles of spindle cells with minimal pleomorphism, admixed with inflammatory cells, mostly lymphocytes, plasma cells, and interspersed collections of neutrophils. (Figures 2B-2D). Infiltration into lung parenchyma could be appreciated in sections (Figure 2B). Focal necrosis and a few scattered mitotic figures were present. A few ganglion-like cells with abundant eosinophilic cytoplasm were also seen.

The patient symptomatically improved after the surgery, and the WBC count reduced gradually reaching 24x10^3^/µL at the end of one month after surgery. After a few days, she developed pain in the back again and CT chest revealed a recurrent growth in the apical space which appeared to invade the right lung (Figure 1C). WBC count was found to be 68x10^3^/µL. She underwent repeat surgery after two months of the first surgery, where an apical tumor adherent to the right lung and anterior chest wall was removed. We received the excised specimen in three parts measuring 12x9.5x5cm, 3x2.5x2.5cm, and 5x3.5x3cm.

**Figure 3:**
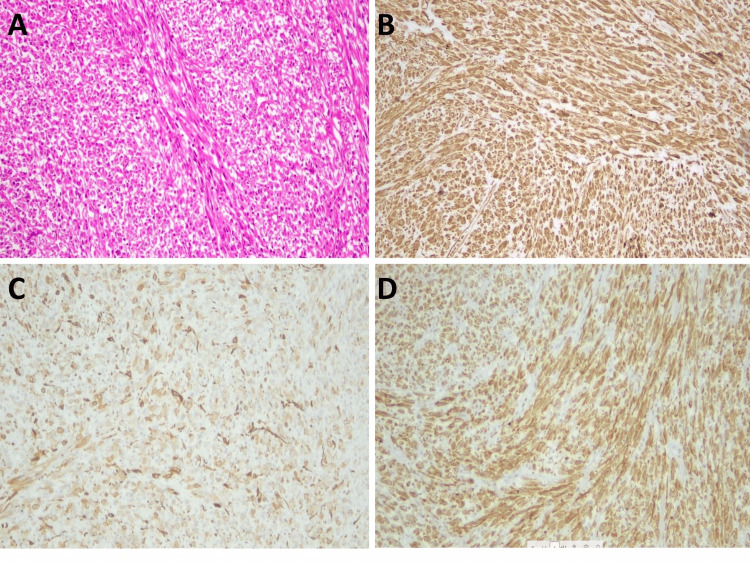
Microscopic and IHC features of the recurrent tumor (A) High power view of tumor on recurrence, H&E, x400. (B) Tumor cells showing vimentin positivity IHC, x400. (C) Tumor cells showing SMA positivity IHC, x400. (D) Tumor cells showing diffuse ALK positivity IHC, x400.

On microscopic examination sections from all of them showed a highly cellular spindle cell tumor with mild nuclear atypia, necrosis, and scattered mitotic figures (Figure 3A). IHC revealed diffuse strong ALK 1 (CD246) positivity along with vimentin and SMA positivity with 20%-30% Ki 67 labeling index (Figures 3B-3D). Morphology and IHC confirmed the diagnosis of recurrent IMT. 

WBC count started falling after the surgery. The patient was started on Crizotinib 250mg BD. At the end of one month of the second surgery, WBC count was found to be 55x10^3^/µL with 92% neutrophils.

**Figure 4:**
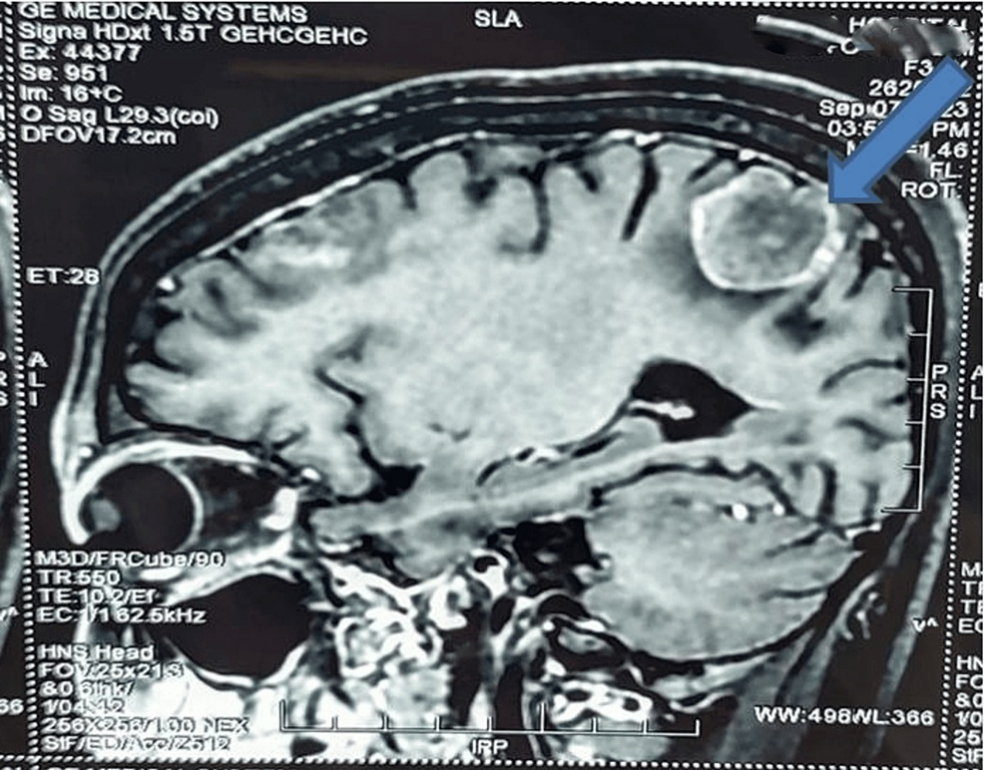
CEMRI image showing metastatic lesion in brain(arrow)

Three months after the second surgery, the patient developed vertebral metastasis. After two months, she developed headaches and vomiting, and a contrast-enhanced MRI (CEMRI) of the brain revealed multiple supratentorial metastatic lesions (Figure 4).

Crizotinib was discontinued, Lorlatinib 100mg OD was started and whole brain radiation was given. There was clinical response in the form of gradual disappearance of symptoms. WBC count came down to 12x10^3^/µL. After one year, CEMRI brain revealed a mild reduction in the size of the lesions and also in mass effect. Lorlatinib was continued as before.

At the time of last follow up, more than two years after initial diagnosis, the patient was found to be with stable disease maintaining a good quality of life. The patient's tolerance to both Crizotinib and Lorlatinib was good without any notable adverse effect.

## Discussion

According to the current World Health Organization of Tumors, IMTs are intermediate-grade, rarely metastasizing neoplasms of soft tissue which have a high recurrence rate after excision and exhibit low metastatic potential [1]. This tumor has always been an enigmatic entity because of its rarity, controversial nomenclature, difficult-to-understand pathogenesis, and unpredictable behavior.

Histomorphologically it comes under the category of inflammatory spindle cell lesions which includes reactive lesions such as inflammatory pseudotumor (IPT) and a plethora of neoplasms - benign, intermediate, and malignant, including many sarcomas. Many different terms have been used to refer to these tumors which include plasma cell granuloma, inflammatory myofibrohistiocytic proliferation, fibroxanthoma, xanthogranuloma, etc. The adjective “inflammatory” and associated leucocytosis may mislead clinicians about its nature and behavior [2].

Although IMTs are said to be common in children and young individuals, they may affect any age [3,4]. IMTs can occur in any location, the common locations being the abdominal cavity (especially in the mesentery, retroperitoneum, and omentum), thoracic cavity including the lungs, areas of the head and neck, urinary bladder, central nervous system, and the female genital tract [1,2]. Symptoms appear due to the tumor mass effect depending upon anatomical location. Our case is an adult female of 35 years with a primary location in the thoracic cavity (lung and mediastinum) having predominantly respiratory symptoms at the time of diagnosis.

It has been noted that IMT can induce inflammation with leukocytosis, neutrophilia, and elevation of CRP and ESR [4]. In our case, marked neutrophilic leukocytosis was a constant companion of the tumor at the time of diagnosis, local recurrence, and metastasis which was a remarkable feature.

The radiological presentation of IMT is heterogeneous, nonspecific, and varies from infiltrating lesion to well-circumscribed mass. In our case, at the outset, the mass was well-circumscribed and was suspected to be a parasitic cyst or neural tumor which are also common in this location.

Histopathological evaluation and IHC are crucial for the diagnosis of IMTs. IMT often presents as a circumscribed, nodular mass; but multinodular lesions are also seen [5]. It has a wide morphological spectrum, which ranges from paucicellular spindle cell proliferation to a highly cellular myofibroblastic proliferation with frankly atypical, neoplastic elements with variable amounts of inflammatory components [4,5]. An exhaustive panel of IHC is required to differentiate IMT from other inflammatory lesions with spindle cells, both benign and malignant e.g. inflammatory fibroid polyp, nodular fasciitis, IPT, desmoids fibromatosis, idiopathic retroperitoneal fibrosis, Gastrointestinal stromal tumor (GIST), inflammatory liposarcoma, inflammatory fibrosarcoma, etc. [2]. IPT, a general term that encompasses reactive mass-forming lesions -infective or autoimmune origin, must not be used for IMT [2]. IMTs usually show reactivity for vimentin and SMA. Positive or negative staining can be observed against other myoid (desmin, H-caldesmon, transgelin), or endometriod markers, Bcl6, interferon-induced membrane protein 1(IFITM1). The tumors do not show reactivity to S100, myogenin, CD117, or epithelial membrane antigen (EMA). Proliferation index Ki 67 is found to be variable. Approximately 50%-60% of IMTs carry ALK gene rearrangement and overexpressed ALK protein can be detected by IHC [1]. However, molecular genetic assays, such as fluorescent in situ hybridization (FISH) and next-generation sequencing (NGS) are required to confirm exact gene rearrangement. In ALK-negative cases, IMT is a diagnosis of exclusion. IHC for ROS1 and/or molecular tests for non-ALK gene fusion may become useful to confirm the diagnosis in ALK-negative cases [1].

In our case, the pathological diagnosis was not difficult because it displayed a conventional morphology with a characteristic hematological picture and ALK positivity. High cellularity, presence of ganglion-like cells, necrosis, and infiltrative border were a few factors present in our case, which are considered to be adverse factors by some authors [5]. According to Han et al., high mitotic activity, hypercellularity, and atypical spindle cells may predict metastatic potential in IMTs irrespective of the ALK status [6].

Rare IMTs with epithelioid morphology, i.e., epithelioid IMTs also known as epithelioid inflammatory myofibroblastic sarcoma (EIMS) behave aggressively as compared to the remarkably indolent behavior of conventional IMT. Malignant IMT is a term used by some authors in locally advanced, recurrent, aggressive, and metastatic diseases with supportive histological features such as increased mitotic activity, high proliferative index (up to 85%), and significant atypia [7,8]. Our case did not fulfill all the criteria to be called malignant.

Distant metastasis is a rare phenomenon in IMTs, and a few cases have been reported in the literature [6]. It is reported to be seen in less than 5% of cases and common sites are lung, liver, and brain. Han et al. reported an incidence of 1.13% in their study of five years. This includes two cases of early metastasis from primary lung IMT to subcutaneous tissue in the neck and multiple subcutaneous and bone metastasis, within a very short period of one and two months [6]. In a study by Irodi et al., 12.5% of cases of thoracic IMT developed brain metastasis on follow-up [9]. Rare cases of the coexistence of pulmonary and brain IMTs have been reported, where it was not clear whether the brain lesion was metastatic or part of multifocal disease [10]. Jehangir et al. suggested that patients with IMT in the lungs should be screened for brain metastasis even if asymptomatic [10]. Our patient developed a rapid local recurrence, followed by spinal and brain metastasis within a short interval of six to eight months.

Treatment of IMT depends on the location and extent of the disease. Surgery remains the treatment of choice for localized tumors. The standard procedure is en block resection with R0 margins, requiring wide local excision in large tumors. After complete excision, although no adjuvant therapy is indicated, adjuvant radiotherapy may have a role in decreasing local recurrence. Systemic treatments are reserved for advanced, non-operable diseases. Several chemotherapeutic agents are being used with variable efficacy but currently, no standard chemotherapy regimen has been established [4].

It has been concluded from detailed genetic studies that IMTs are oncogene-driven, approximately half of them are ALK-positive harboring ALK fusion gene, and the other half are ALK negative and could harbor other fusion genes or mutations such as ROS1, NTRK3, RET, and platelet-derived growth factor beta (PDGFB) [1]. This revelation opened up the possibility of effective use of targeted therapy in this tumor. A first-generation tyrosine kinase inhibitor (TKI), Crizotinib, an inhibitor of ALK, MET, and ROS 1, is now in use as targeted therapy. European Organisation for Research and Treatment of Cancer (EORTC) 90101 (CREATE) trial confirmed that crizotinib is effective, with durable responses, in patients with locally advanced or metastatic ALK-positive IMT [11]. In patients developing Crizotinib resistance, second- and third-generation ALK inhibitors with increased ALK selectivity such as ceritinib, alectinib, brigatinib, lorlatinib, and ensartinib are reported to show variable response [4].

ALK inhibitors are found to be effective in temporary prevention of disease progression. Resistance and recurrence after the use of third-generation ALK inhibitors have been reported [12]. After local recurrence, our patient was treated with crizotinib and then switched to lorlatinib, when she developed crizotinib resistance, with brain metastasis. Lorlatinib, a third-generation ALK/ROS 1 inhibitor, has a higher potency in ALK inhibition and CNS penetration.

In this case, there was a challenge in the diagnosis of the disease initially, however, because of conventional morphology and ALK positivity, the pathological diagnosis was not difficult. Complete surgical resection of the tumor was a challenge because of its location and proximity to vital structures. Finally, resistance to TKI leading to disease progression in the form of metastasis was a major challenge in the management of the disease in this case.

## Conclusions

IMT is said to be a rare tumor of intermediate malignancy with low metastatic potential, common in children and young adult. IMT in adults with conventional morphology and ALK positivity with rapid local recurrence and distant metastasis is rarely reported. Because of its seemingly inflammatory and reactive morphological features, one may miss the diagnosis, especially in small biopsy. A high index of suspicion and detailed IHC and preferably a genetic study are needed for its diagnosis.

Genetic analysis and targeted therapy with ALK-TKIs have come to the forefront in the treatment of advanced, unresectable, recurrent, and metastatic IMTs. However, loss of efficacy due to the development of resistance is the main obstacle. This case gave us an opportunity to study all aspects of this rare enigmatic tumor.
